# Peroxiredoxin 2 Ameliorates AβO-Mediated Autophagy by Inhibiting ROS via the ROS–NRF2–p62 Pathway in N2a-APP Swedish Cells

**DOI:** 10.3390/antiox11101889

**Published:** 2022-09-23

**Authors:** Wei Jin, Min Kyoung Kam, Sung Woo Lee, Young-Ho Park, Hong Jun Lee, Dong-Seok Lee

**Affiliations:** 1 School of Life Sciences, BK21 FOUR KNU Creative BioResearch Group, Kyungpook National University, Daegu 41566, Korea; 2 School of Life Sciences & Biotechnology, College of Natural Sciences, Kyungpook National University, Daegu 41566, Korea; 3 Futuristic Animal Resource & Research Center (FARRC), Korea Research Institute of Bioscience and Biotechnology (KRIBB), Cheongju 28116, Korea; 4 College of Medicine and Medical Research Institute, Chungbuk National University, Cheongju 28644, Korea; 5 Research Institute eBiogen Inc., Seoul 04785, Korea

**Keywords:** Alzheimer’s disease, oxidative stress, autophagy, ROS–NRF2–p62 pathway, cell death, peroxiredoxin 2, N2a-APPswe cells

## Abstract

In Alzheimer’s disease, reactive oxygen species (ROS) are generated by the deposition of amyloid-beta oligomers (AβOs), which represent one of the important causes of neuronal cell death. Additionally, AβOs are known to induce autophagy via ROS induction. Previous studies have shown that autophagy upregulation aggravates neuronal cell death. In this study, the effects of peroxiredoxin 2 (Prx2), a member of the peroxidase family of antioxidant enzymes, on regulating AβO-mediated autophagy were investigated. Prx2 decreased AβO-mediated oxidative stress and autophagy in N2a-APPswe cells. Further, we examined the relationship between the neuronal protective effect of Prx2 and a decrease in autophagy. Similar to the effects of N-acetyl cysteine, Prx2 decreased AβO-induced ROS and inhibited p62 protein expression levels by downregulating the activation of NRF2 and its translocation to the nucleus. In addition, treatment with 3-methyladenine, an autophagy inhibitor, ameliorates neuronal cell death. Overall, these results demonstrate that the Prx2-induced decrease in autophagy was associated with the inhibition of ROS via the ROS–NRF2–p62 pathway in N2a-APPswe cells. Therefore, our results revealed that Prx2 is a potential therapeutic target in anti-Alzheimer therapy.

## 1. Introduction

Alzheimer’s disease (AD) is the most common neurodegenerative disease, affecting millions of people worldwide. Typical pathological features of AD include the deposition of β-amyloid plaques due to the accumulation of amyloid-beta oligomers (AβO) [1], which is generated by the sequential proteolytic cleavage of transmembrane amyloid precursor protein (APP) by β-secretases and γ-secretases, which are considered critical initiators of AD [2,3]. Reactive oxygen species (ROS) are small and highly reactive molecules that are generated by the accumulation of AβOs [4] and can oxidize proteins, lipids, and DNA [5]. ROS-mediated oxidative stress leads to cell death [6,7] and induces autophagy [5,7]. Autophagy is an essential, conserved, self-eating process that maintains cellular homeostasis. Autophagy begins at the phagophore and ends with autophagosomal death. Phagophore formation is controlled by multiple signaling events, such as ATG proteins and Beclin-1. Lc3 (microtubule-associated protein 1A/1B-light chain 3) is a soluble protein widely distributed in mammalian tissues and cultured cells. During autophagy, Lc3 is processed and recruited into the extending phagophore [8,9,10]. SQSTM/p62 is an autophagosome cargo protein that recognizes toxic cellular waste and transfers it to autophagosomes [11,12]. It has been reported that ROS can initiate autophagosome formation and autophagic degradation as cellular signaling molecules [7,13]. Autophagy involves clearing aggregated proteins and damaged organelles to reduce oxidative stress [14]. In contrast, several studies have suggested that autophagy might have negative effects, as well, and that excessive autophagy results in cell death, which is called autophagic cell death. Autophagic cell death is characterized by the accumulation of autophagosomes and large autolysosomes in which whole cell contents are engulfed and digested [6,15]. However, little is known about the molecular mechanisms underlying AβO-mediated oxidative stress and autophagy in AD.

Peroxiredoxins (Prxs) are part of the peroxidase family, and they have a large and highly conserved cysteine residue, designated the “peroxidatic” Cys (C_p_). Prxs reduce hydrogen peroxide, organic hydroperoxides, and peroxynitrite. Therefore, Prx enzymatic activity is important for protecting cellular components from oxidative stress [16,17,18]. According to recent studies, under oxidative stress, autophagy and antioxidant proteins, such as peroxiredoxin, stabilize ROS levels to maintain the redox status [7,19]. Prx2 has been shown to play an important role in antioxidant activity and exert protective effects against cytotoxicity and neurodegeneration [20,21,22,23]. However, further research on the relationship between Prx2 and autophagy associated with AD is required.

Here, we explored whether Prx2 overexpression reduces AβO-mediated oxidative stress and, ultimately, ameliorates neuronal cell death. Furthermore, we investigated the molecular mechanism linking Prx2 and autophagy in N2a-APP Swedish (N2a-APPswe) cells.

## 2. Materials and Methods

### 2.1. Chemicals and Reagents

N-acetyl cysteine (NAC), chloroquine (CQ), rapamycin, and 3-methyladenine (3-MA) were purchased from Sigma-Aldrich (St. Louise, MO, USA). CM-H2DCFDA was purchased from Thermo Fisher Scientific (Waltham, MA, USA). A CytoTox 96 Non-Radioactive Cytotoxicity Assay kit was purchased from Promega Corporation (Madison, WI, USA). A CCK cell viability assay kit was purchased from Donginbiotech (Seoul, Korea). A MycoAlert Mycoplasma Detection Kit was purchased from Lonza (Rockland, ME, USA).

### 2.2. Cell Culture

The N2a mouse neuroblastoma cell line (ATCC, Manassas, VA, USA) was used in this study. The N2a cells were cultured in minimal essential medium (MEM; Welgene, Daegu, Korea) containing 10% fetal bovine serum (FBS; Thermo Fisher Scientific, Waltham, MA, USA) and 1% penicillin (Welgene, Daegu, Korea) at 37 °C in 5% CO_2_. The N2a cells were passaged using 0.05% trypsin (Welgene, Daegu, Korea) twice a week and used between passages 15 to 25. The medium was changed daily. The mycoplasma was detected using the MycoAlert Mycoplasma Detection Kit (Lonza, Rockland, ME, USA), according to the manufacture’s protocol (Figure S1).

### 2.3. Plasmid Construction

The APP gene and Prx2 gene were obtained from Addgene (Boston, MA, UAS). The APPswe mutation gene was introduced using the Site-Directed Mutagenesis kit (Stratagene, La Jolla, CA, USA) according to the manufacture’s protocol. The *APPswe* and *Prx2* genes were amplified by polymerase chain reaction (PCR) using LA Taq polymerase (TaKaRa, Shiga, Japan). These genes were cloned into pCR8/GW/TOPO (Thermo Fisher Scientific) and inserted into the pLenti6.3/v5-DEST vector (Thermo Fisher Scientific) using the LR clonase (Thermo Fisher Scientific). Construct sequences were confirmed using DNA sequencing. The following primers were used: APP (Forward: 5′-CGGAGGAGATCTCTGAAGTGAATCTGGATGCAGAATTCCGA-3′, Reverse: 5′-TCGGAATTCTGCATCCAGATTCACTTCAGAGATCTCCTCCG-3′) and Prx2 (Forward: 5′- ATGGCCTCCGGTAACGCG-3′, Reverse: 5′-ATTGTGTTTGGAGAAATATTCC-3′). 

### 2.4. Transfection and Preparation of Stable Cell Lines

The N2a cells (1 × 10^5^) were seeded in 6-well plates and cultured for 24 h. Plenti6.3-APPswe plasmid (1 μg) was transfected into the N2a cells using Effectene (Qiagen, Valencia, CA, USA), according to the manufacturer’s instructions. After 9 h, cells were washed with PBS and incubated in fresh medium overnight. After transfection for a day, the N2a-APPswe cells were selected using 8 μg/mL blasticidin (Thermo Fisher Scientific). As for the generation of the N2a-APPswe-Prx2 cells, the Prx2-expressing cells were generated from the N2a-APPswe cells through transfection with the pLenti6.3-Prx2 plasmid (1 μg) using Effectene.

### 2.5. Measurement of Intracellular ROS Generation

The production of intracellular ROS was measured using the ROS-specific fluorescent dye CM-H2DCFDA (Thermo Fisher Scientific). The cells were seeded at an initial density of 1 × 10^6^ cells in 60 Φ plates and cultured for 24 h. The cells were then washed with phosphate-buffered saline (PBS), followed by the addition of 1 mL Dulbecco’s modified Eagle’s medium (DMEM; Welgene, Daegu, Korea) (Phenol Red free media) containing 2.5 μM CM-H2DCFDA and incubated for 15 min at 37 °C in the dark. The ROS levels were analyzed using a flow cytometer (BD Biosciences, Franklin Lakes, NJ, USA).

### 2.6. Measurement of AβO-Induced Cytotoxicity

AβO-induced damage to the plasma membranes of the N2a cells was quantified by the amount of lactate dehydrogenase (LDH) released using the CytoTox 96 Non-Radioactive Cytotoxicity Assay kit (Promega Corporation, Madison, WI, USA). The cells were plated at a density of 1 × 10^6^ cells in 60 Φ plates and cultured for 24 h. A total of 50 μL of culture medium was added to 96-well plates, followed by 50 μL of assay reagent. The LDH activity in the culture medium was measured by monitoring the absorbance at 490 nm.

### 2.7. Immunocytochemistry

The N2a, N2a-APPswe, and N2a-APPswe-Prx2 cells were seeded on round coverslips for 24 h and then treated with CQ (25 μM) for 24 h. The cells were fixed with 5% paraformaldehyde and permeabilized with 0.25% Triton X-100 in PBS (PBST) for 10 min. The cells were then treated with 1% bovine serum albumin in PBST and incubated overnight at 4 °C with an anti-Lc3 antibody diluted in a blocking solution. After washing three times with PBS, the cells were treated with Alexa Fluor 647 goat anti-rabbit secondary antibody for 4 h at room temperature. The cells were washed three times with PBS, coverslips were mounted on slides using VECTASHIELD medium (Vector Laboratories, Burlingame, CA, USA), and images were obtained using a Carl Zeiss LSM-710 confocal microscope (Carl Zeiss, Oberkochen, Germany).

### 2.8. RNA Extraction and Reverse Transcription-Polymerase Chain Reaction (RT-PCR)

The total RNA was extracted from the cells cultured for 24 h using TRIzol solution (Bio Science Technology, Korea), following the manufacturer’s protocol. Complementary DNA was synthesized using total RNA (1 μg) and AccuPower RT-PCR Premix (Bioneer, Daejeon, Korea) (1 μg). The NRF2, p62, and GAPDH (control) [24] cDNA fragments were amplified using specific primers. The primer sequences used were mouse GAPDH (Forward: 5′-ACCACAGTCCATGCCATCAC-3′, Reverse: 5′-TCCACCACCCTGTTGCTGTA-3′), mouse p62 (Forward: 5′-CCTCAGCCCTCTAGGCATTG-3′, Reverse: 5′-CCGGGGATCAGCCTCTGTAG-3′), and mouse NRF2 (Forward: 5′-CAGCCAGCTGACCTCCTTAG-3′, Reverse: 5′-GGGATTCACGCATAGGAGCA-3′). The cDNA was synthesized in the following conditions: one cycle of denaturing at 94 °C (5 min), 20–25 cycles of denaturing at 94 °C (45 s), annealing at 60 °C (45 s), elongation at 72 °C (45 s), and one cycle of final elongation at 72 °C (5 min).

### 2.9. Isolation of the Nuclear and Cytoplasmic Proteins

The nuclear and cytoplasmic proteins were isolated from the N2a, N2a-APPswe, and N2a-APPswe-Prx2 cells cultured for 24 h using the NE-PER Nuclear and Cytoplasmic Extraction Reagent kit (Thermo Fisher Scientific), following the manufacturer’s protocol.

### 2.10. Western Blot Analysis

The cells were harvested using a scraper (SPL Life Science Co., Pocheon, Korea) and lysed using the ice-cold PRO-PREP protein extraction solution (iNtRON Biotechnology Inc., Korea). Protein quantification was performed using the Bradford assay (Bio-Rad, Hercules, CA, USA), and 10–15 µg of protein lysates were separated by electrophoresis on 6–15% SDS-PAGE and then transferred onto nitrocellulose membranes (Pall Corporation, New York, NY, USA). The membranes were blocked with 5% skim milk (BD Biosciences) and incubated with primary antibodies overnight at 4 °C. The primary antibodies used were anti-Aβ (6E10) (1: 1000) and anti-APP (1: 1000) (Abcam, Cambridge, MA, USA), anti-ATG5 (1: 1500), anti-ATG7 (1: 1500), anti-Lc3 (1: 2000), anti-p62 (1: 2000), anti-PARP (1: 2000), anti-cleaved caspase-3 (1: 1000), anti-keap1 (1: 2000) (Cell Signaling Technology, Danvers, MA, USA), anti-β-actin (1: 5000), anti-GAPDH (1: 5000), anti-NRF2 (1: 2000), anti-HO-1 (1: 2000) (Santa Cruz Biotechnology, Santa Cruz, CA, USA), anti-lamin B1 (1: 2000) (Thermo Fisher Scientific), anti-Beclin-1 (1: 1000), anti-Prx2 (1: 2000) (AbFrontier, Seoul, Korea), and anti-v5 (1:1 500) (Invitrogen, Carlsbad, CA, USA). The membranes were washed five times with the Tris-buffered saline Tween 20 (TBST) for 5 min and then incubated with horseradish peroxidase-conjugated goat anti-rabbit (1: 5000) (Cell Signaling Technology) and anti-mouse (1: 5000) (Thermo Fisher Scientific) secondary antibodies overnight at 4 °C. The excess secondary antibodies were removed by washing six times with TBST. Specific binding was detected using Clarity Western ECL substrate (Bio-Rad), according to the manufacturer’s instructions.

### 2.11. Cell Viability Assay 

The cells were plated at a density of 1 × 10^6^ cells in 60 Φ plates and cultured for 24 h. A 50 μL/mL CCK cell viability assay kit (Donginbiotech, Seoul, Korea) was added for 30 min at 37 °C. Then, a 200 μL culture medium was added to the 96-well plates. The cell viability was measured by monitoring the absorbance at 450 nm.

### 2.12. Statistical Analysis

Statistical analyses were performed using the GraphPad Prism software (San Diego, CA, USA). Data are presented as the means ± standard errors of the means (SEM) from at least three independent experiments (n ≥ 3). Statistically significant differences were determined using one-way ANOVA. Asterisks indicate calculated *p* values as follows: * *p* < 0.05, ** *p* < 0.01, and *** *p* < 0.001. The significance threshold was set at *p* < 0.05.

## 3. Results

### 3.1. Accumulation of AβO Increased the Levels of ROS, Cytotoxicity, and Cell Death in N2a APPswe Cells

**Figure 1:**
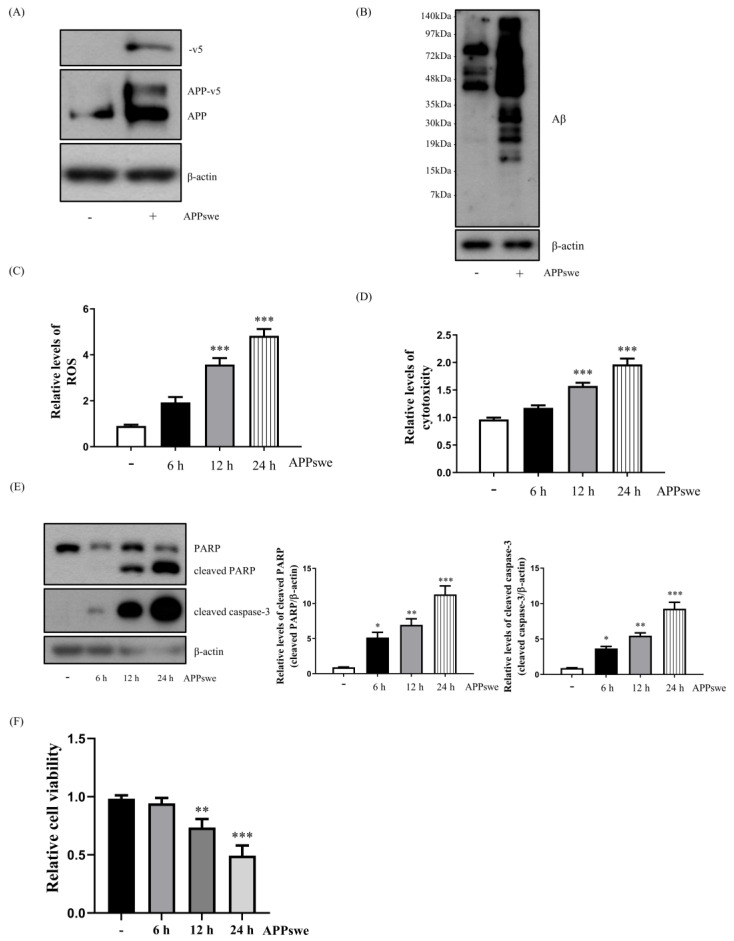
Amyloid-beta oligomer (AβO) accumulation increases the levels of reactive oxygen species (ROS), cytotoxicity, and cell death in N2a-APPswe cells. (**A**) Western blot analysis showing the APPswe gene’s exogenous transduction in the N2a and APPswe stably expressing N2a (N2a-APPswe) cells. (**B**) Amyloid-beta oligomer expression was evaluated by Western blot analysis in N2a and N2a-APPswe cells. (**C**) Flow cytometry analysis of intracellular ROS levels in N2a-APPswe cells cultured for 6, 12, and 24 h, measured using the fluorescent dye CM-H2DCFDA (2.5 μM). (**D**) Lactate dehydrogenase (LDH) analysis of cytotoxicity in N2a-APPswe cells cultured for 6, 12, and 24 h. (**E**) Western blot analysis of poly (ADP-ribose), polymerase (PARP), and cleaved caspase-3 expression in N2a-APPswe cells cultured for 6, 12, and 24 h. (**F**) Cell viability was measured using a CCK cell viability assay kit for N2a-APPswe cells cultured for 6, 12, and 24 h. Data are represented as the means ± SEMs of three independent experiments (* *p* < 0.05, ** *p* < 0.01, and *** *p* < 0.001).

To analyze the relationship between Prx2 and autophagy during AβO accumulation, we used the N2a cells that stably expressed the APP Swedish mutation (N2a-APPswe), where AβO accumulation was increased due to abnormal β- and γ-secretase cleavage. First, we confirmed that the APPswe was stably expressed and accumulation of AβO was higher in the N2a-APPswe cells (Figure 1A,B). Next, we investigated whether the levels of ROS and cytotoxicity, as well as those of the cleaved poly (ADP-ribose), polymerase (PARP), caspase-3 (cell death markers), and cell viability, increased in a time-dependent manner in the N2a-APPswe cells (Figure 1C–F). Our results suggest that the AβO accumulation increased oxidative stress and cytotoxicity.

### 3.2. Autophagy Was Increased by APP Swedish Mutation

**Figure 2:**
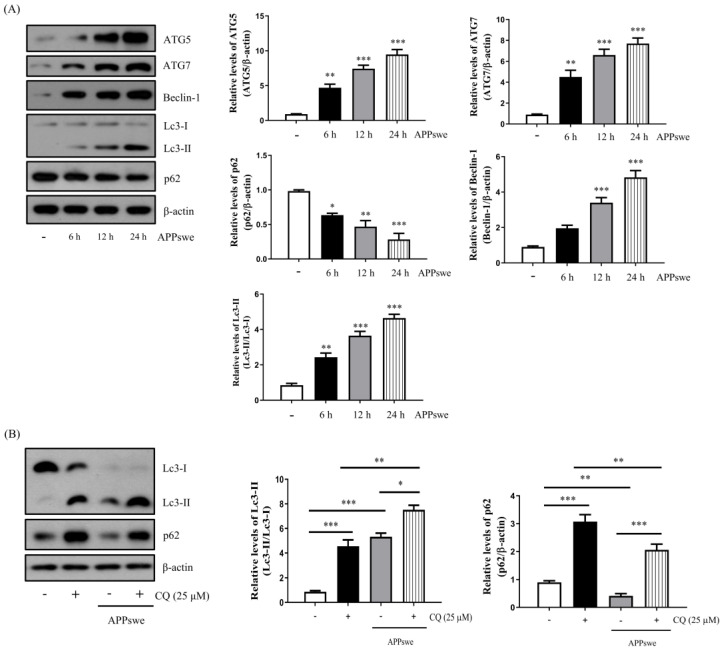

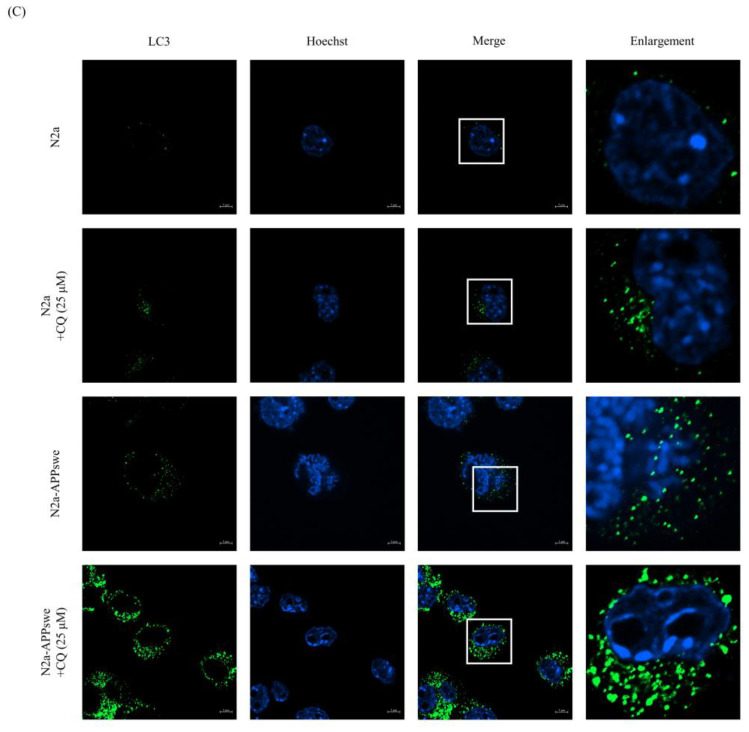
Autophagy was increased by APP Swedish mutation in N2a-APPswe cells. (**A**) Western blot analysis of ATG5, ATG7, Beclin-1, Lc3, and p62 expression in N2a-APPswe cells cultured for 6, 12, and 24 h. (**B**) Lc3 and p62 protein expression was determined by Western blot analysis using N2a-APPswe cells cultured for 24 h in the presence or absence of chloroquine (CQ) (25 μM) treatment for 24 h. (**C**) Confocal microscopy analysis of Lc3 puncta in N2a and N2a-APPswe cells cultured for 24 h in the presence or absence of CQ (25 μM) treatment for 24 h. Data are represented as the means ± SEMs of three independent experiments (* *p* < 0.05, ** *p* < 0.01, and *** *p* < 0.001).

It has been reported that autophagy can be induced by ROS [5,7] and that an increase in autophagy is observed in the early stages of AD [25]. Therefore, we measured the autophagy marker proteins ATG5, ATG7, Beclin-1 (initiator of autophagy), Lc3 (autophagosome membrane extension and fusion participation protein), and p62 (autophagic degradation target cargo protein) levels. The results showed that the ATG5, ATG7, Beclin-1, and the Lc3-II/Lc3-I ratios increased, while the ratio of p62 decreased, in a time-dependent manner in the N2a-APPswe cells (Figure 2A). 

To determine the autophagic flux of the N2a-APPswe cells, we treated the cells with 25 μM CQ, an autophagy inhibitor that suppresses autophagosome-lysosome fusion and decreases autophagic degradation [26]. As shown in Figure 2B, autophagy was increased by APPswe and the levels of Lc3-II/Lc3-I and p62 were higher in the CQ treatment group than in the no-treatment group (Figure 2B). Confocal microscopy images showed that the number of Lc3 puncta in the N2a-APPswe cells was higher than that in the N2a cells. Furthermore, we observed that the number of Lc3 puncta was increased in the N2a-APPswe cells compared to that in the CQ-treated group (Figure 2C). Thus, we suggest that autophagy increases in a time-dependent manner in N2a-APPswe cells.

### 3.3. Autophagy and Cell Death Were Increased by AβO-Mediated Oxidative Stress

**Figure 3:**
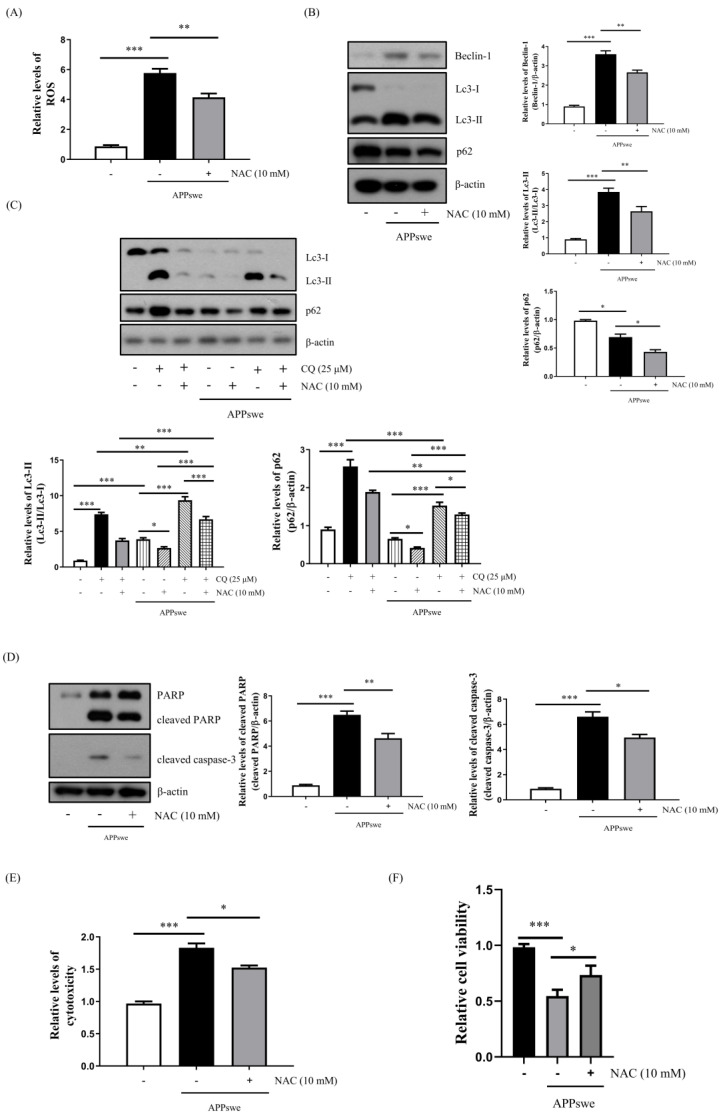
Autophagy, cytotoxicity, and cell death were increased by AβO-mediated oxidative stress in N2a-APPswe cells. (**A**) Intracellular ROS levels were determined using flow cytometry in N2a and N2a-APPswe cells cultured for 24 h and treated with N-acetylcysteine (NAC) (10 mM) for 24 h. (**B**) Western blot analysis of Beclin-1, Lc3, and p62 proteins from N2a and N2a-APPswe cells cultured for 24 h and treated with NAC (10 mM) for 24 h. (**C**) Lc3 and p62 protein expression was determined by Western blot analysis using N2a and N2a-APPswe cells cultured for 24 h with or without CQ (25 μM) and/or NAC (10 mM) for 24 h. (**D**) PARP and cleaved caspase-3 protein levels were analyzed by Western blot analysis in N2a and N2a-APPswe cells cultured for 24 h with NAC (10 mM). (**E**) LDH analysis of cytotoxicity in N2a and N2a-APPswe cells cultured for 24 h with NAC (10 mM). (**F**) Cell viability was measured using a CCK cell viability assay kit for N2a and N2a-APPswe cells cultured for 24 h and treated with NAC (10 mM) for 24 h. Data are represented as the means ± SEMs of three independent experiments (* *p* < 0.05, ** *p* < 0.01, and *** *p* < 0.001).

To confirm whether AβO-mediated ROS generation increased autophagy, we used N-acetylcysteine (NAC), which is widely used as an antioxidant reagent [27]. Following treatment with 10 mM NAC, the levels of ROS (Figure 3A) and autophagy were significantly decreased. Interestingly, p62 protein levels were also decreased (Figure 3B). To investigate whether NAC treatment decreased p62 protein levels during the autophagic flux, we co-treated cells with CQ. As shown in Figure 3C, the expression levels of the Lc3-II/Lc3-I and p62 proteins were increased following the CQ treatment, while those of p62 were decreased by the NAC treatment. In addition, we discovered that cell death and cytotoxicity were ameliorated by the NAC treatment (Figure 3D–F). Thus, we suggest that AβO-mediated oxidative stress increases autophagy and cell death. 

### 3.4. Overexpression of Prx2 Decreased ROS Levels and Autophagy

**Figure 4:**
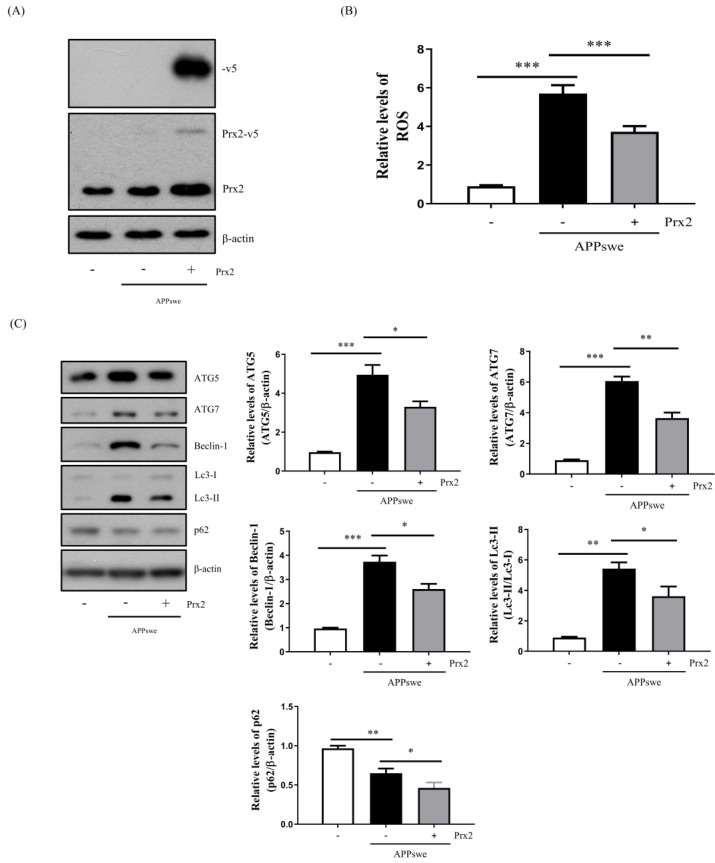

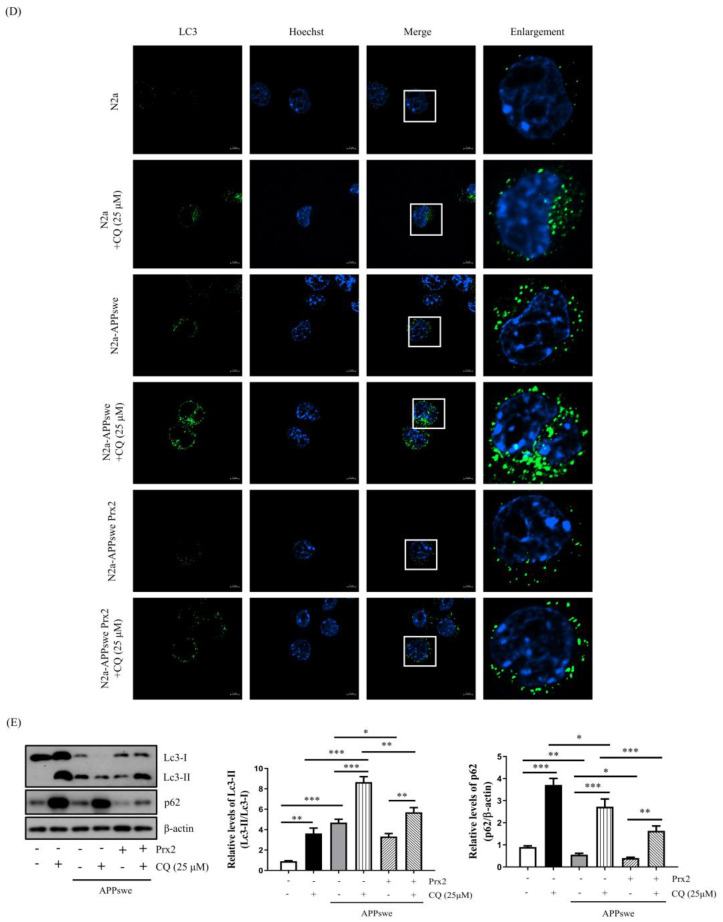
Peroxiredoxin (Prx) 2 overexpression decreased the levels of ROS and autophagy in N2a-APPswe cells. (**A**) The expression of Prx2 and –v5 was confirmed by Western blot analysis in N2a, N2a-APPswe, and N2a-APPswe-Prx2 cells cultured for 24 h. (**B**) Flow cytometry analysis of the intracellular ROS levels in N2a, N2a-APPswe, and N2a-APPswe-Prx2 cells cultured for 24 h. (**C**) ATG5, ATG7, Beclin-1, Lc3, and p62 protein expression levels were analyzed by Western blot analysis in N2a, N2a-APPswe, and N2a-APPswe-Prx2 cells cultured for 24 h. (**D**) Confocal microscopy analysis of Lc3 puncta in N2a, N2a-APPswe, and N2a-APPswe-Prx2 cells cultured for 24 h with or without CQ (25 μM). (**E**) Lc3 and p62 protein expression levels were determined by Western blot analysis in N2a, N2a-APPswe, and N2a-APPswe-Prx2 cells cultured for 24 h with or without CQ (25 μM). Data are represented as the means ± SEMs of three independent experiments (* *p* < 0.05, ** *p* < 0.01, and *** *p* < 0.001).

Prxs have been reported to exert antioxidant effects [17,18]. Furthermore, Prx2 exhibits excellent antioxidant and neuronal protective effects in neurodegenerative diseases [22,23]. To investigate whether Prx2 has a neuroprotective effect in N2a-APPswe cells, we generated N2a-APP-Prx2 cells transfected with the pLenti6.3-Prx2 vector (Figure 4A). We discovered that the ROS- and AβO-mediated autophagy levels were decreased by Prx2 overexpression in the N2a-APPswe cells (Figure 4B,C). Interestingly, the p62 protein levels were also decreased (Figure 4C). Using confocal microscopy, we measured the Lc3 puncta and marked their decrease following Prx2 overexpression in the N2a-APPswe cells (Figure 4D). Western blot analysis also showed that the Lc3-II/Lc3-I and p62 levels were decreased by Prx2 overexpression, regardless of the presence or absence of CQ (Figure 4E). Thus, we suggest that Prx2 decreases AβO-mediated ROS production, which ultimately reduces autophagy in N2a-APPswe cells.

### 3.5. Overexpression of Prx2 Downregulated p62 Transcription 

**Figure 5:**
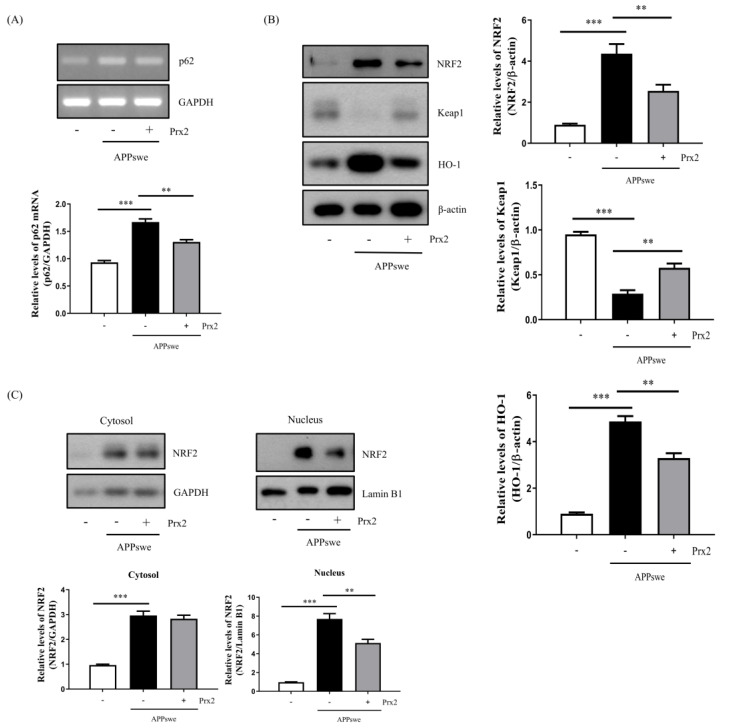
Prx2 overexpression decreased autophagy via the ROS–NRF2–p62 pathway in N2a-APPswe cells. (**A**) The p62 mRNA levels were analyzed using RT-PCR in N2a, N2a-APPswe, and N2a-APPswe-Prx2 cells cultured for 24 h. (**B**,**C**) Western blot analysis of NRF2, Keap1, and HO-1 in the whole cell lysate, nucleus, or cytosol of N2a, N2a-APPswe, and N2a-APPswe-Prx2 cells cultured for 24 h. Data are represented as the means ± SEMs of three independent experiments (** *p* < 0.01, and *** *p* < 0.001).

According to a previous study, a decrease in autophagy is accompanied by a decrease in LC3-II, as well as an increase in p62 protein, which is less degraded [11]. However, as shown in Figure 3B and Figure 4C, the ROS reduction induced by Prx2 overexpression and NAC treatment did not decrease the expression of p62 protein in autophagosomes. To analyze the transcriptional levels of p62, we used RT-PCR and measured the p62 mRNA levels. First, we confirmed that the NAC treatment downregulated p62 transcription in N2a-APPswe cells (Figure S2). In N2a-APPswe-Prx2 cells, p62 transcription was also downregulated (Figure 5A). Therefore, we suggest that ROS reduction due to Prx2 overexpression downregulates p62 transcription.

### 3.6. Overexpression of Prx2 Decreased Autophagy via the ROS–NRF2–p62 Pathway

NRF2, which belongs to the basic leucine zipper (bZIP) family of transcription factors, responds to oxidative stress. Under oxidative stress, NRF2 is isolated from Keap1 and translocated to the nucleus to activate the antioxidant response elements (ARE), such as heme oxygenase (HO-1), located in the p62 promoter region, which ultimately upregulates the expression of p62 mRNA [7,28,29]. Therefore, we investigated whether Prx2 decreases autophagy via the ROS–NRF2–p62 pathway. We discovered that NRF2 and HO-1 were increased, while Keap1 expression was decreased, in N2a-APPswe cells. In contrast, Prx2 overexpression decreased NRF2, as well as HO-1, and increased Keap1 (Figure 5B). In addition, as shown in Figure 5C, in N2a-APPswe cells, NRF2 was significantly increased in the nucleus, while Prx2 overexpression decreased its translocation. Therefore, these results indicate that Prx2 reduces AβO-mediated oxidative stress by suppressing NRF2 activation and translocation to the nucleus. Prx2 decreases ROS levels to inhibit the ROS-NRF2-p62 pathway, which plays an important role in the oxidative stress pathway associated with the autophagy process.

### 3.7. Overexpression of Prx2 Ameliorated Cytotoxicity and Cell Death in N2a-APPswe Cells

**Figure 6:**
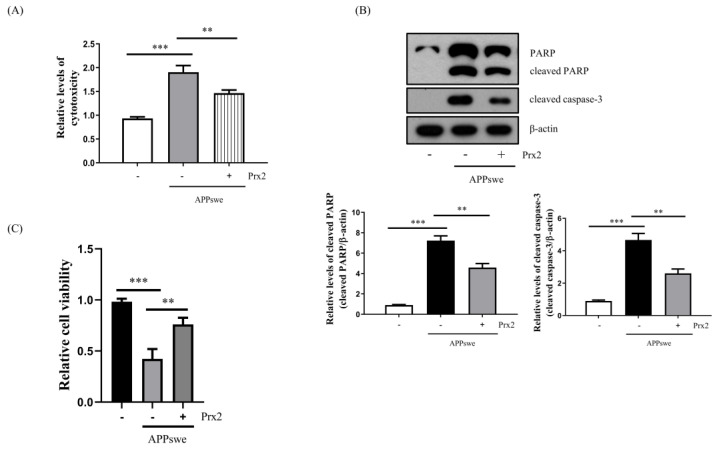
Prx2 overexpression decreased cytotoxicity and cell death in N2a-APPswe cells. (**A**) LDH analysis of cytotoxicity in N2a, N2a-APPswe, and N2a-APPswe-Prx2 cells cultured for 24 h. (**B**) Western blot analysis of PARP and cleaved caspase-3 protein expression levels in N2a, N2a-APPswe, and N2a-APPswe-Prx2 cells cultured for 24 h. (**C**) Cell viability was measured using a CCK cell viability assay kit for N2a, N2a-APPswe and N2a-APPswe-Prx2 cells cultured for 24 h. Data are represented as the means ± SEMs of three independent experiments (** *p* < 0.01, and *** *p* < 0.001).

Finally, we investigated the neuroprotective effect of Prx2 overexpression against AβO accumulation in N2a-APPswe cells. The results showed that Prx2 overexpression decreased cytotoxicity and cell death in N2a-APPswe cells (Figure 6). In addition, to determine whether AβO-mediated autophagy can lead to cell death, we used 3-MA, which inhibits autophagy by targeting PI3K [30]. In the N2a-APPswe cells, autophagy was inhibited following treatment with 1 mM 3-MA for 4 h (Figure S3A). Furthermore, we observed that cell death was attenuated (Figure S3B,C). In contrast, we found that treatment with 50 nM rapamycin for 24 h, which is an autophagy inducer, aggravated cell death during autophagy induction in the N2a-APPswe cells (Figure S3D–F). Taken together, these results suggest that AβO-mediated autophagy can lead to cell death in N2a-APPswe cells.

## 4. Discussion

Autophagy is the process of maintaining cellular homeostasis in response to diverse stress conditions, such as starvation, brain injury, and genotoxic stress [7]. Recently, several studies have reported that oxidative stress acts as the converging point of these stimuli with ROS, which induces autophagy among the main intracellular signal transducers [5,7,15]. ROS are small and highly reactive molecules that can be generated by AβO accumulation [4]. Numerous studies have shown that autophagy is decreased in AD models due to dysfunctional autophagy [31,32,33]. However, an increase in autophagy has also been reported in the early stages of AD [25]. Therefore, we investigated whether AβO-induced autophagy played a role in neuronal cell death. In our study, we first confirmed that autophagy was increased in a time-dependent manner by the accumulation of AβO in the N2a-APPswe cells (Figure 2A). To determine whether the increased autophagy and cell death were due to an APPswe mutation, we generated N2a-Mock cells as a sham control group (Figure S4). As shown in Figure 2B,C, the autophagic flux of the N2a-APPswe cells was blocked by the CQ treatment. In contrast, the number of Lc3 puncta in the N2a-APPswe cells was higher than that in the N2a cells. Furthermore, the NAC treatments decreased AβO-mediated autophagy and apoptosis (Figure 3B–F). These results indicate that NAC, an antioxidant reagent, decreases autophagy and apoptosis following AβO-induced oxidative stress. 

The Prx family of antioxidant enzymes scavenges peroxides via the cysteine residue in its active site. In particular, our previous studies have shown that Prxs exhibit antitoxic and antioxidant effects on AβO in neuronal cells [34,35]. Furthermore, it has been reported that Prx2 also exhibits antioxidant and neuroprotective effects [22,36]. Therefore, we investigated the relationship between Prx2 and AβO-mediated autophagy. Upon the overexpression of Prx2, we observed a decrease in the ROS levels and AβO-mediated autophagy (Figure 4B,C). Furthermore, Prx2 overexpression attenuated AβO-mediated cell death and cytotoxicity (Figure 6), indicating that Prx2 has a protective effect against AβO-mediated oxidative stress and toxicity. 

According to recent studies, ROS-induced autophagy is meant to clear ROS and maintain cellular homeostasis, whereas excess ROS-induced autophagy also increases the generation of ROS and aggravates cell death [6,15,37]. In this study, we observed that a decrease in cell death was accompanied by a reduction in autophagy (Figure 3B,D, Figure 4C and Figure 6B,C). To confirm whether AβO-mediated autophagy could lead to cell death, we treated cells with 3-MA, an autophagy inhibitor [30], and rapamycin, an autophagy inducer. Our results showed that cell death was attenuated in the N2a-APPswe cells when autophagy was inhibited and that it was aggravated during autophagy induction (Figure S3). Furthermore, it also has been reported that decreased autophagy can protect neurons from Aβ [38,39,40]. Thus, we suggest that AβO-mediated autophagy aggravates cell death in N2a-APPswe cells. The regulation of autophagy may delay the onset of AD, which makes it a viable pathway that can be pharmacologically targeted.

In autophagy-related studies, decreased levels of autophagy are accompanied by an increase in the level of p62, which is a cargo receptor protein usually degraded by autophagosomes that plays a transfer role in autophagy [10,11]. Interestingly, we discovered that decreased autophagy was accompanied by a decrease in p62 expression levels following the NAC treatment (Figure 3B). Prx2 overexpression also supported this finding (Figure 4C). Under stressed conditions, increased NRF2 protein can enter the nucleus to induce the transcription of p62 through the NRF2 non-canonical pathway [28,41]. Therefore, we conducted further research on p62-related pathways. The NRF2–p62 pathway has been revealed as part of the relationship between the antioxidant response and autophagy [12,42]. In normal cellular conditions, NRF2, an antioxidant transcription factor, is bound to Keap1, an E3 ubiquitin ligase adaptor protein, and it leads to NRF2 ubiquitination and degradation by the proteasome. Under oxidative stress, Keap1 is challenged to undergo critical cysteine modifications and blocks the ubiquitylation of NRF2, which allows Keap1 to bind p62 and degrade it via autophagy (called the NRF2 non-canonical pathway) [12]. Our results showed that the AβO-mediated ROS production increased NRF2 protein levels and decreased Keap1 protein levels (Figure 4B and Figure 5B). Further, our results revealed that the NRF2 nucleoprotein levels were much higher than those in the cytoplasm in the N2a-APPswe cells (Figure 5C). Alternatively, other studies have shown that p62 can also induce NRF2 [28,42], which is in line with our analysis of NRF2 mRNA levels in the N2a-APPswe cells. We confirmed that NRF2 mRNA levels were also increased compared to those in the control group (Figure S5). Further, we showed that Prx2 overexpression induced a decrease in ROS levels, which reduced the accumulation of NRF2 protein and downregulated the transcription of p62 (Figure 4A–C and Figure 5). These results indicate that AβO-mediated autophagy is induced by the ROS-induced increase in p62, the transcription of which is induced by the nucleoprotein NRF2. Here, we showed that Prx2 ameliorated AβO-mediated autophagy by inhibiting ROS via the ROS–NRF2–P62 pathway in the N2a-APPswe cells. 

## 5. Conclusions

**Figure 7:**
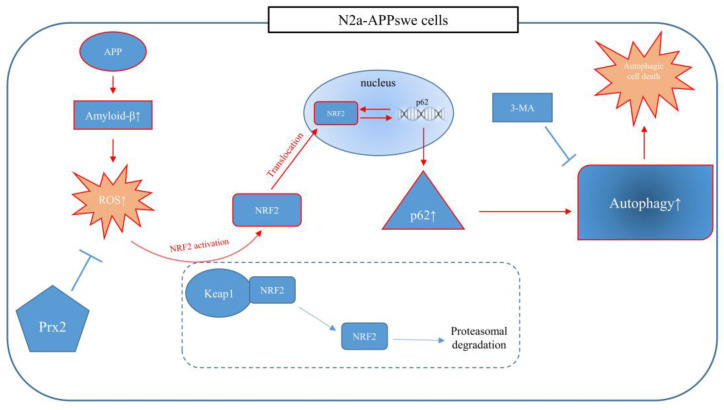
A schematic diagram demonstrates that Prx2 decreases AβO-mediated autophagy by inhibiting ROS via the ROS-NRF2-p62 pathway and ameliorates AβO-mediated autophagic cell death.

ROS induces autophagy by isolating NRF2 from Keap1 and translocating NRF2 to the nucleus, which ultimately promotes p62 transcription. In contrast, the overexpression of Prx2 decreased AβO-mediated autophagy by inhibiting ROS generation to suppress NRF2 translation and p62 transcription. Therefore, for the first time, we showed that Prx2 overexpression decreased AβO-mediated autophagy and reduced cell death via the ROS-NRF2-p62 pathway in N2a-APPswe cells (Figure 7). We believe that autophagy regulation may delay the onset of AD, and that Prx2 might represent an efficient antioxidant for neuronal protection, which makes it a viable pharmacological target.

## Data Availability

The data is contained within the article or in the Supplementary Materials.

## Supplementary Materials

The following supporting information can be downloaded at: https://www.mdpi.com/article/10.3390/antiox11101889/s1, Figure S1. Mycoplasma was not detected in N2a, N2a-APPswe and N2a-APPswe-Prx2 cells. Figure S2. The transcription of p62 was downregulated by NAC in N2a-APPswe cells. Figure S3. AβO-mediated autophagy aggravated cell death in N2a-APPswe cells. Figure S4. The increase of cell death and autophagy dues to APPswe mutation. Figure S5. NRF2 transcription was decreased by Prx2 overexpression in N2a-APPswe cells.
