# Supplementary material for: Peroxiredoxin 2 Ameliorates AβO-Mediated Autophagy by Inhibiting ROS via the ROS–NRF2–p62 Pathway in N2a-APP Swedish Cells
Source: Antioxidants (Basel). 2022 Sep 23;11(10):1889. doi: 10.3390/antiox11101889 (PMC9598687; doi:10.3390/antiox11101889)
Supplement: Supplementary file 1 [file antioxidants-11-01889-s001.zip › antioxidants-1911327-supplementary.pdf]

antioxidants

MDPI

Supplementary Materials: Peroxiredoxin 2 Ameliorates AβO-Mediated Autophagy by Inhibiting ROS via the ROS–NRF2–p62 Pathway in N2a-APP Swedish Cells

Wei Jin [1,2], Min Kyoung Kam [1,2], Sung Woo Lee [1,2], Young-Ho Park [3], Hong Jun Lee [4,5] and Dong-Seok Lee [1,2,*]

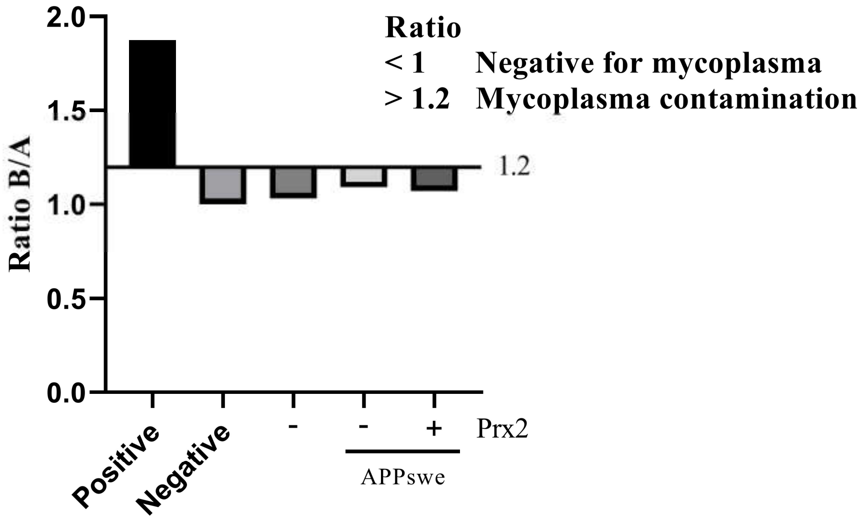

Figure S1. The mycoplasma was detected using MycoAlert Mycoplasma Detection Kit in N2a, N2a-APPswe and N2a-APPswe-Prx2 cells cultured for 24h, positive control (MycoAlert Assay Control Sets) and Negative control (deionized water) were used.

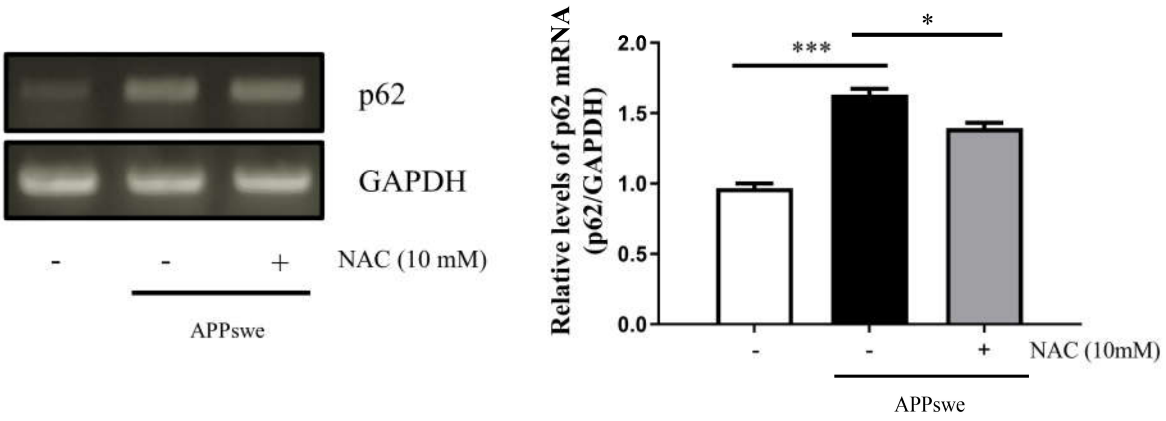

Figure S2. The p62 mRNA levels were determined using Reverse transcription-polymerase chain reaction (RT-PCR) in N2a and N2a-APPswe cells cultured for 24 h in the presence or absence of N-acetyl cysteine (NAC) (10 mM) for 24 h. Data are represented as the mean ± SEM of three independent experiments (*$p < 0.05$, **$p < 0.01$, ***$p < 0.001$).

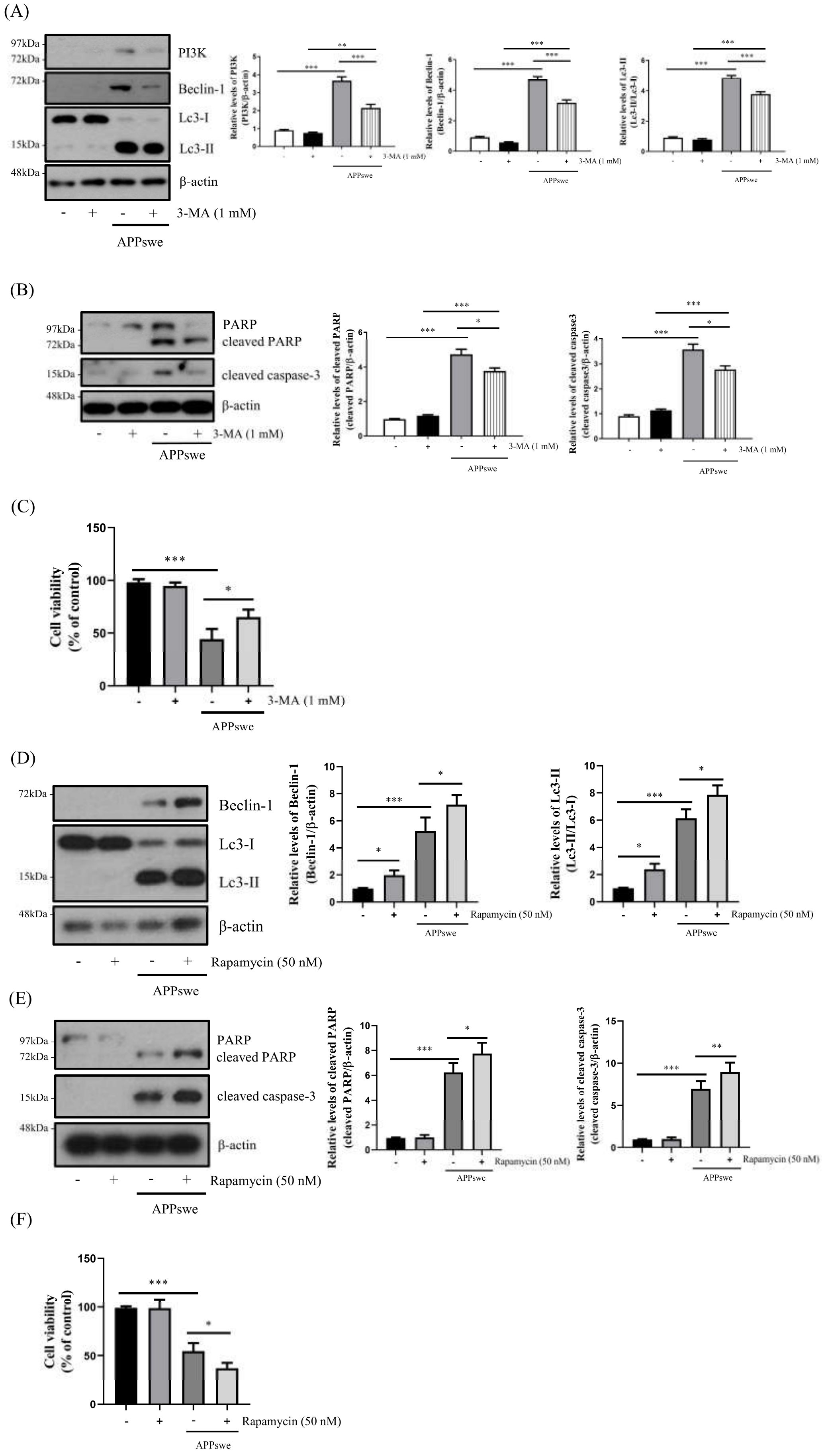

(A)
97kDa
72kDa
PI3K
72kDa
Beclin-1
Lc3-I
15kDa
Lc3-II
48kDa
β-actin
- + - + 3-MA (1 mM)
APPswe
Relative levels of PI3K (PI3K/β-actin)
Relative levels of Beclin-1 (Beclin-1/β-actin)
Relative levels of Lc3-II (Lc3-II/Lc3-I)
(B)
97kDa
72kDa
PARP
cleaved PARP
15kDa
cleaved caspase-3
48kDa
β-actin
- + - + 3-MA (1 mM)
APPswe
Relative levels of cleaved PARP (cleaved PARP/β-actin)
Relative levels of cleaved caspase3 (cleaved caspase3/β-actin)
(C)
Cell viability (% of control)
- + - + 3-MA (1 mM)
APPswe
(D)
72kDa
Beclin-1
Lc3-I
15kDa
Lc3-II
48kDa
β-actin
- + - + Rapamycin (50 nM)
APPswe
Relative levels of Beclin-1 (Beclin-1/β-actin)
Relative levels of Lc3-II (Lc3-II/Lc3-I)
(E)
97kDa
72kDa
PARP
cleaved PARP
15kDa
cleaved caspase-3
48kDa
β-actin
- + - + Rapamycin (50 nM)
APPswe
Relative levels of cleaved PARP (cleaved PARP/β-actin)
Relative levels of cleaved caspase-3 (cleaved caspase-3/β-actin)
(F)
Cell viability (% of control)
- + - + Rapamycin (50 nM)
APPswe

Figure S3. (A) Western blot analysis of PI3K, Beclin-1, and Lc3 protein levels in N2a and N2a-APPswe cells cultured for 24 h with or without a 4-h treatment with 3-methyladenine (3-MA) (1 mM). (B) PARP and cleaved caspase-3 protein levels were determined by western blotting in N2a and N2a-APPswe cells cultured for 24 h with or without 3-MA (1 mM) for 4 h. (C) Cell viability was measured by using CCK cell viability assay kit in N2a and N2a-APPswe cells cultured for 24 h with or without 3-MA (1 mM) for 4 h. (D, E) Expression of Beclin-1, Lc3, PARP and cleaved caspase-3 was determined by western blotting in N2a and N2a-APPswe cells cultured for 24 h with or without rapamycin (50 nM) for 24 h. (F) Cell viability was measured by using CCK cell viability assay kit in N2a and N2a-APPswe cells cultured for 24 h with or without rapamycin (50 nM) for 24 h. Data are represented as the mean ± SEM of three independent experiments ($^{*}p < 0.05$, $^{**}p < 0.01$, $^{***}p < 0.001$).

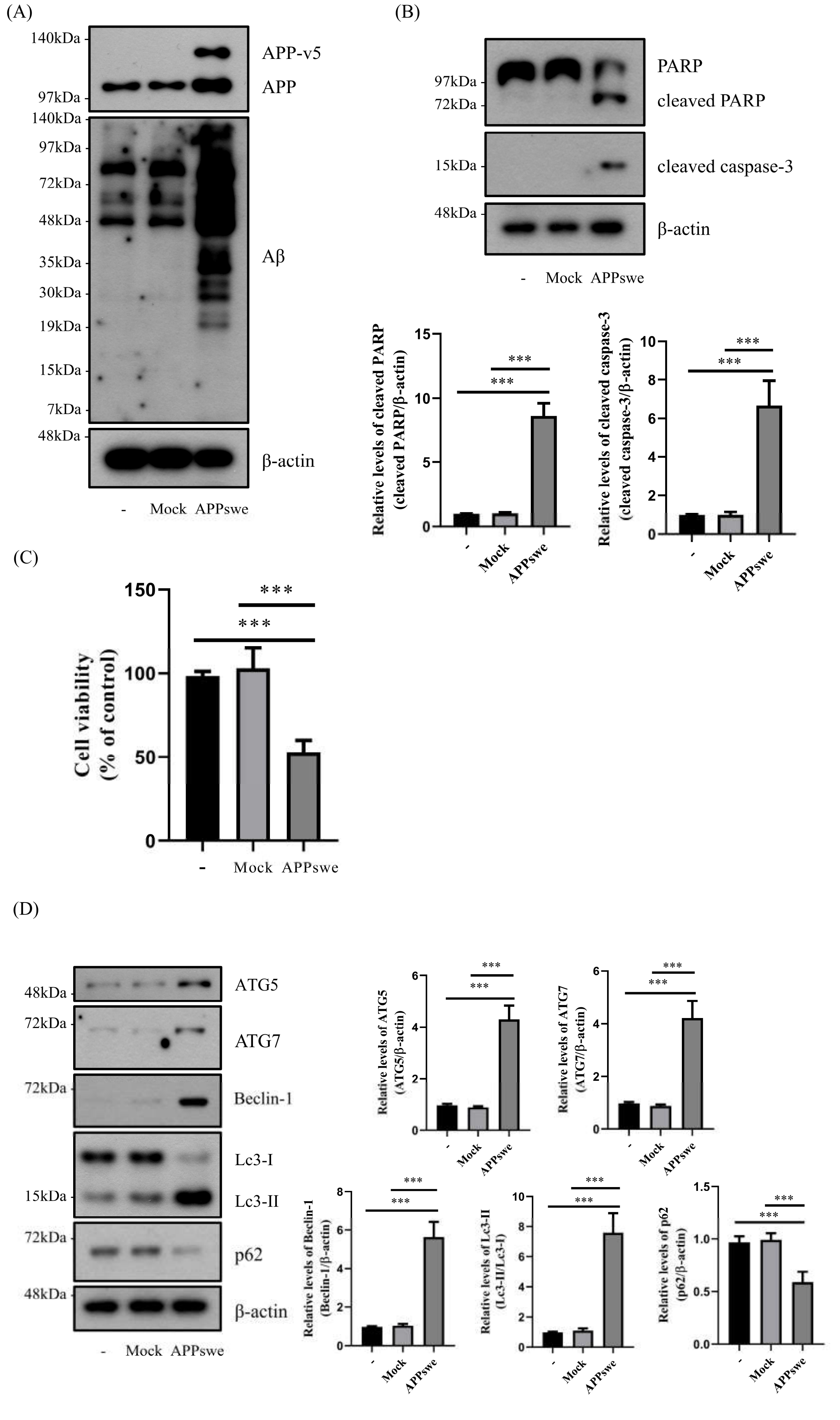
(A)
140kDa
97kDa
APP-v5
APP
140kDa
97kDa
72kDa
48kDa
35kDa
30kDa
19kDa
15kDa
7kDa
Aβ
48kDa
β-actin
- Mock APPswe
(B)
97kDa
72kDa
PARP
cleaved PARP
15kDa
cleaved caspase-3
48kDa
β-actin
- Mock APPswe
Relative levels of cleaved PARP (cleaved PARP/β-actin)
Relative levels of cleaved caspase-3 (cleaved caspase-3/β-actin)
***
(C)
Cell viability (% of control)
- Mock APPswe
(D)
ATG5
ATG7
Beclin-1
Lc3-I
Lc3-II
p62
β-actin
48kDa
72kDa
15kDa
Relative levels of ATG5 (ATG5/β-actin)
Relative levels of ATG7 (ATG7/β-actin)
Relative levels of Beclin-1 (Beclin-1/β-actin)
Relative levels of Lc3-II (Lc3-II/Lc3-I)
Relative levels of p62 (p62/β-actin)

Figure S4. (A, B) Expression of APP, Aβ, PARP and cleaved caspase-3 was determined by western blotting in N2a, N2a-LacZ (Mock) and N2a-APPswe cells cultured for 24h. (C) Cell viability was measured by using CCK cell viability assay kit in N2a, N2a-LacZ (Mock) and N2a-APPswe cells cultured for 24h. (D) Western blot analysis showing ATG5, ATG7, Beclin-1, Lc3, and p62 expression in N2a, N2a-LacZ (Mock) and N2a-APPswe cells cultured for 24h. Data are represented as the mean ± SEM of three independent experiments (*$p < 0.05$, **$p < 0.01$, ***$p < 0.001$).

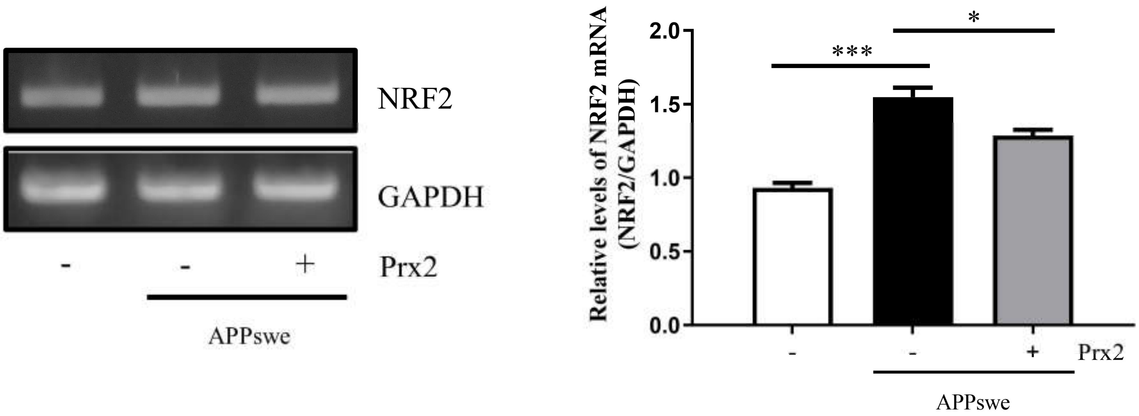

Figure S5. NRF2 mRNA levels were assessed using RT-PCR in N2a, N2a-APPswe, and N2a-APPswe-Prx2 cells cultured for 24 h. Data are represented as the mean ± SEM of three independent experiments (*$p < 0.05$, **$p < 0.01$, ***$p < 0.001$).
